# Prenatal PCB-153 Exposure and Decreased Birth Weight: The Role of Gestational Weight Gain

**DOI:** 10.1289/ehp.1307796

**Published:** 2014-04-01

**Authors:** Eva Govarts, Maribel Casas, Greet Schoeters, Merete Eggesbø, Damaskini Valvi, Mark Nieuwenhuijsen, Jens Peter Bonde, on behalf of the ENRIECO, OBELIX, CHICOS Consortia

**Affiliations:** 1 Environmental Risk and Health, Flemish Institute for Technological Research (VITO), Mol, Belgium; 2 Center for Research in Environmental Epidemiology (CREAL), Barcelona, Spain; 3 Department of Genes and Environment, Norwegian Institute of Public Health, Oslo, Norway; 4 Department of Occupational and Environmental Medicine, Copenhagen University Hospital Bispebjerg, Copenhagen, Denmark

Verner et al. (2013) recently questioned whether the association between cord levels of PCB-153 (polychlorinated biphenyl-153) and birth weight reported in our recent meta-analysis (Govarts et al. 2012) might be attributable to the influence of gestational weight gain (GWG), a factor that may be related to both PCB-153 concentrations and birth weight. By using simulated data in a physiologically based pharmacokinetic (PBPK) model, Verner et al. (2013) concluded that

The association between prenatal levels of PCBs and birth weight may be strongly confounded by the effect of [GWG] on both blood PCB levels and birth weight.

In our recently published meta-analysis (Govarts et al. 2012), the influence of weight gain was considered only in a sensitivity analysis because we did not have this information from many cohorts at that time. Since the publication of that article, we have obtained more individual-level data on GWG for a number of the cohorts, allowing us to repeat these analyses to further assess such influence.

Seven of the 12 birth cohorts that participated in our previous meta-analysis (Govarts et al. 2012) had data on GWG available, for a total of 4,473 mother–child pairs. GWG data from 3 cohorts was extracted from prenatal records, whereas for the other cohorts the classification was based on mother’s recall. In most cohorts, weight in late pregnancy was obtained at birth or at some weeks before delivery, but in two cohorts these data were obtained some years after birth. In two cohorts, both measured and self-reported data on mother’s weight was available, and an excellent correlation was observed (Spearman *r* = 0.95; *p* < 0.001). In the present reanalysis of the data, we also estimated the changes in fat mass (FM) as a function of GWG (Butte et al. 2003). Spearman correlation coefficients were calculated between PCB-153 and GWG/FM and between GWG/FM and birth weight. We first estimated the association between cord serum PCB-153 and birth weight using linear multivariate-adjusted regression models in each cohort separately, and then we used mixed-effect models to estimate the combined effect (pooled analysis). We included absolute GWG or estimated FM as additional potential confounders in the models.

The absolute GWG and estimated FM distributions were relatively similar among cohorts (mean + SD was 13.9 ± 5.3 kg for GWG and 5.5 ± 4.5 kg for FM). We found low correlations between PCB-153 and GWG/FM and between GWG/FM and birth weight (*r* < 0.18). The effect estimate of the combined analysis yielded a negative association between PCB-153 and birth weight equivalent to a 293-g reduction [95% confidence interval (CI): –536, –50] in birth weight per 1-µg/L increase in cord serum PCB-153. After including absolute GWG or estimated FM in the model, the strength of the association was reduced by 48% [–153 g (95% CI: –340, 30)] and 31% [–204 g (95% CI: –411, 0)], respectively.

Our reanalysis of earlier data with some new data confirms that GWG may indeed confound the relationship between PCB-153 and birth weight; however, when we included in the model either absolute GWG or estimated FM, which is a more precise estimate of maternal lipid gain (Butte et al. 2003), we still observed an important reduction in birth weight as PCB-153 levels increased. We cannot exclude the possibility of residual confounding due to potential measurement error of GWG, which may further reduce the effect toward the null. However, two of the included cohorts showed high correlations between measured and self-reported weight, suggesting that misclassification due to maternal weight reports is likely to be small and negligible. Misclassification could result from inaccurate assessment of FM. In a recent study, Maple-Brown et al. (2013) observed that maternal age and prepregnancy body mass index can be related to an increase in gestational FM, suggesting that it is worth considering both of them in models assessing fat mass.

As suggested by Verner et al. (2013), we have considered GWG as a potential confounder because it increases the volume of lipids diluting PCB levels in blood (Glynn et al. 2011) and increases birth weight of the offspring (Siega-Riz et al. 2009). However, other than a confounding factor, GWG could be an intermediate factor on a causal path between PCB-153 exposure and birth weight. If this is the case, then controlling for GWG in the models would lead to an overadjustment bias (Schisterman et al. 2009) in the effect estimates and would be inappropriate if the main aim is to estimate the total (and not the direct) effect of PCB-153 on birth weight.

In conclusion, in interpreting the findings by Verner et al. (2013) one should take into account that PBPK models, although useful, are based on many assumptions. The only way to verify them is through testing in population-based studies. Our analysis clearly indicates that attributing all the effect of PCBs on birth weight to GWG appears an oversimplification.
